# Pro-Calcitonin, Erythrocyte Sedimentation Rate and C - reactive Protein in Predicting Diabetic Foot Ulcer Characteristics; a Cross Sectional Study

**Published:** 2019-07-10

**Authors:** Fahimeh Hadavand, Atefeh Amouzegar, Hessam Amid

**Affiliations:** 1 Infectious Diseases and Tropical Medicine Research Center, Shahid Beheshti University of Medical Sciences, Tehran, Iran.; 2 Department of Nephrology, Firoozgar Clinical Research Development Center (FCRDC), Iran University of Medical Sciences, Tehran, Iran.

**Keywords:** blood sedimentation, procalcitonin, diabetic foot, peripheral arterial disease

## Abstract

**Introduction:**

Considering the importance of early diagnosis of diabetic foot ulcers and its complications, this study aimed to evaluate the accuracy of erythrocyte sedimentation rate (ESR), C - reactive protein (CRP), and pro-calcitonin (PCT) in predicting the ulcer class, osteomyelitis, and peripheral arterial disease (PAD).

**Methods:**

This cross-sectional study was performed on 200 consecutive patients suffering from diabetic foot ulcer who were referred to Infectious Disease Ward. The levels of PCT, ESR, and CRP were measured for all patients and the screening performance characteristics of each marker in predicting the ulcer class, osteomyelitis, and PAD was calculated.

**Results:**

The levels of PCT, ESR and CRP were significantly higher in patients with class IV foot ulcer compared to those with class III ulcers (p<0.001). Patients with evidence of osteomyelitis had significantly higher level of PCT, ESR and CRP. The best cutoff points of PCT, ESR and CRP in predicting osteomyelitis were 0.35 ng/ml (86.1% sensitivity, 45.3% specificity), 56.5 mm/hours (95.8% sensitivity, and 50.0% specificity) and 44 mg/ml (90.3% sensitivity, 57.0% specificity), respectively. The presence of PAD was significantly associated with increased levels of the three biomarkers. The best cutoff values for PCT, ESR and CRP in predicting PAD were 0.45 (70.8% sensitivity, 71.7% specificity), 61.5 (83.3% sensitivity, 52.0% specificity) and 49 (83.3% sensitivity, 63.8% specificity), respectively.

**Conclusion:**

Based on the findings of the present study, although the accuracy of PCT, ESR, and CRP in predicting the severity of diabetic foot ulcers was fair, increase in the three parameters can predict the occurrence of osteomyelitis and PAD following diabetic food development with good accuracy and acceptable sensitivity.

## Introduction

Diabetes and its complications have affected a large number of people around the world, with an estimated increased worldwide prevalence in all age groups from 2.8% in 2000 to 4.4% in 2030 (1). Despite the medical and surgical advances in the last decade, problems with diabetic foot, which is one of the most important chronic complications of diabetes, remain a health problem and is the most important cause of non-traumatic foot injuries (2). The likelihood of a diabetic patient developing foot lesions (ulcer / gangrene) is estimated to be 15-25 percent throughout life, with an annual incidence of 1 to 4.1 percent, more than 15 percent of which ultimately results in amputation of the organ. Some risk factors of diabetic foot ulcers are as follow: having had diabetes for over 10 years, male gender, uncontrolled blood sugar, and cardiovascular, renal and ocular diseases (2). Another important issue in patients with diabetic foot ulcers is the high susceptibility of these ulcers to severe inflammation and wound infections. Patients with diabetes mellitus are prone to severe foot infection due to neuropathy and vascular dysfunction (3). 

It is difficult to determine severity of diabetic foot ulcer and to distinguish non-infectious and infectious wounds in the early stages (4). When clinical symptoms are misleading, laboratory tests can help determine disease severity and also diagnose the foot ulcer infection. 

Many studies have focused on the role of inflammatory markers such as erythrocyte sedimentation rate (ESR) and C - reactive protein (CRP), as well as pro-calcitonin (PCT) to predict the onset of inflammation and infection, especially in bacterial involvement of diabetic wounds (5-8). In addition, the diagnostic and predictive values of some circulating inflammatory parameters such as leukocyte counts and circulating inflammatory proteins are evaluated (9, 10).

PCT is a protein precursor of calcitonin hormone, which is synthesized and secreted by the C-cells in the thyroid gland. PCT production has been proven to occur after activation of inflammatory cascade by the liver and mononuclear cells and is modulated through cytokines associated with sepsis (11). PCT has also been shown to play a major role in diagnosis of diabetic foot ulcers, and its role can be even more important than CRP (12-14). Based on above-mentioned points, this study aimed to evaluate the value of PCT, ESR, and CRP in predicting the ulcer class, as well as presence or absence of osteomyelitis and peripheral arterial disease (PAD).

## Methods

***Study design and settings***

This cross-sectional study was performed on 200 consecutive patients suffering from diabetic foot ulcer who were referred to Infectious Disease ward of Imam Hussein Hospital, Tehran, Iran, from January 2017 to September 2018. The levels of PCT ESR, and CRP were measured for all patients and the screening performance characteristics of each marker in predicting the ulcer class, osteomyelitis, and PAD was calculated. Informed consent was obtained from each patient and the study protocol as approved by the ethics committee of Shahid Beheshti University of Medical Sciences (Ethics code: IR.SBMU.MSP.REC.1396.844)

***Participants***

Adult (age > 18 years) diabetic patients with foot infection, who were hospitalized in the mentioned hospital were included. Patients with septic shock, unstable hemodynamics or without consent to participate in the study were excluded.

***Data gathering***

The baseline characteristics including demographics, medical history, comorbidities, duration of diabetes, and the presence of underlying immunodeficiency or using immunosuppressive medication were collected by reviewing the patients’ profiles. Baseline levels of laboratory biomarkers were also measured and documented for all patients at the time of admission to infectious disease ward. 

Serum PCT was measured using an enzyme-linked fluorescence assay and its concentration was measured using routine immunoassay (BRAHMS PCT KRYPTOR system, BRAHMS, Hennigsdorf, Germany). 

CRP concentration was measured with routine immunoassay (bionic, America). The sensitivity of the assay and the target value of the calibrator have been standardized against the reference material ERM-DA 472/IFCC. ESR was measured using a closed automated method, using a highly sensitive assay.

An infectious disease resident was responsible for data gathering.

***Definitions***

The severity of diabetic foot was classified according to the Perfusion, Extent, Depth, Infection, Sensation (PEDIS) classification system (15). Based on this classification diabetic ulcers are classified as uninfected (grade 1), mild (grade 2), moderate (grade 3), and severe (grade 4). 

Mild ulcers: presence of >2 manifestations of inflammation (purulence, pain, warmth)

Moderate ulcers: cellulitis extending >2 cm and involvement of muscle, tendon, joint

Severe ulcers: infection with systemic toxicity (fever, tachycardia, hypotension, etc.)

Osteomyelitis was diagnosed using magnetic resonance imaging (MRI) findings.

Diabetic foot ulcers were diagnosed based on the clinical manifestation.

PAD was verified based on the ankle brachial index (ABI < 1.2). 

***Statistical analysis***

Descriptive analysis was used to describe the data, including mean ± standard deviation (SD) for quantitative variables and frequency (percentage) for categorical variables. Chi square test, independent t test and Mann-Whitney U test were used for comparison of variables. The correlation between quantitative variables was tested by the Pearson's correlation test. The receiver operating characteristic (ROC) curve analysis was used to assess the value of biomarkers in predicting the studied endpoints. For statistical analysis, the statistical software IBM SPSS Statistics for Windows version 22.0 (IBM Corp. Released 2013, Armonk, New York) was used. P values <0.05 were considered statistically significant.

## Results

***Baseline characteristics of studied patients***

200 patients with the mean age of 61.26 ± 11.32 (33 -87) years were included (71.5% male). According to the PEDIS classification, 45.0% were class III and 55.0% had class IV of diabetic foot ulcer. The mean duration of suffering from diabetes was 11.79 ± 3.94 years. 13.0% were immune-deficient all of whom used immunosuppressive medications. According to the MRI findings, the evidence of osteomyelitis was found in 36.0%. Mean hemoglobin A_1_C was 7.91 ± 1.81% ad it ranged from 4.7 to 18.3. Mean ABI was 1.02 ± 0.22 and 24.0% suffered from PAD with ABI less than 0.9. Regarding laboratory parameters, mean CRP was 51.05 ± 28.30 mg/ml, mean PCT was 0.52 ± 0.47 ng/ml and mean ESR was 66.84 ± 21.96 mm/hr. There was a significant correlation between PCT and ESR (r = 0.395, p < 0.001) as well as PCT and CRP (r = 0.334, p < 0.001).

**Table 1:** Mean pro-calcitonin (PCT), erythrocyte sedimentation rate (ESR), and C - reactive protein (CRP) measures based on ulcer class and presence or absence of osteomyelitis and peripheral arterial disease (PAD)

**Index **	**PCT**	**P**	**CRP**	**P**	**ESR**	**P**
**Ulcer class**						
III	0.39 ± 0.17	<0.001	43.84 ± 24.01	<0.001	59.30 ± 19.81	<0.001
IV	0.63 ± 0.59		56.96 ± 30.22		73.00 ± 21.79	
**Osteomyelitis **						
Absent	0.38 ± 0.17	<0.001	39.35 ± 17.87	<0.001	55.73 ± 15.54	<0.001
Present	0.77 ± 0.69		71.86 ± 31.42		86.57 ± 17.41	
**PAD**						
Absent	0.47 ± 0.48	<0.001	43.68 ± 20.10	<0.001	61.36 ± 19.36	<0.001
Present	0.69 ± 0.39		74.40 ± 36.93		84.17 ± 20.86	

Data are presented as mean ± standard deviation.

**Table 2:** Screening performance characteristics of pro-calcitonin (PCT), erythrocyte sedimentation rate (ESR), and C - reactive protein (CRP) in predicting ulcer class, osteomyelitis, and peripheral arterial disease (PAD)

**Index **	**AUC**	**sensitivity**	**specificity**	**PPV**	**NPV**
**Ulcer class**					
PCT	0.68 (0.60-0.75)	74.54(66.28-82.72)	55.65(51.26-59.89)	67.2	64.1
CRP	0.64 (0.56-0.72)	65.57(61.04-69.73)	46.78(41.75-51.83)	60.0	52.5
ESR	0.66 (0.59-0.74)	63.68(60.76-66.57)	52.28(44.65-59.83)	62.0	54.0
**Osteomyelitis **					
PCT	0.79 (0.72-0.85)	86.17(75.46-94.71)	45.37(39.94-50.73)	47.0	85.3
CRP	0.91 (0.86-0.95)	90.38(80.41-97.27)	57.04(49.08-68.46)	54.1	91.3
ESR	0.87 (0.82-0.92)	95.82(93.89-97.73)	50.08(42.32-57.86)	51.9	95.9
**PAD**					
PCT	0.73(0.64-0.82)	70.88(65.95-76.93)	71.76(69.19-74.17)	44.1	88.6
CRP	0.79(0.71-0.87)	83.36(78.81-87.89)	63.89(58.26-68.13)	42.1	92.4
ESR	0.81(0.73-0.88)	83.32(78.54-87.99)	52.08(47.82-57.19)	35.4	90.8

Data are calculated in cutoffs of 0.35 for PCT, 44.0 for CRP, and 61.5 for ESR. All data are presented with 95% confidence interval. PPV: positive predictive value; NPV: negative predictive value; AUC: area under the ROC curve.

**Figure 1:**
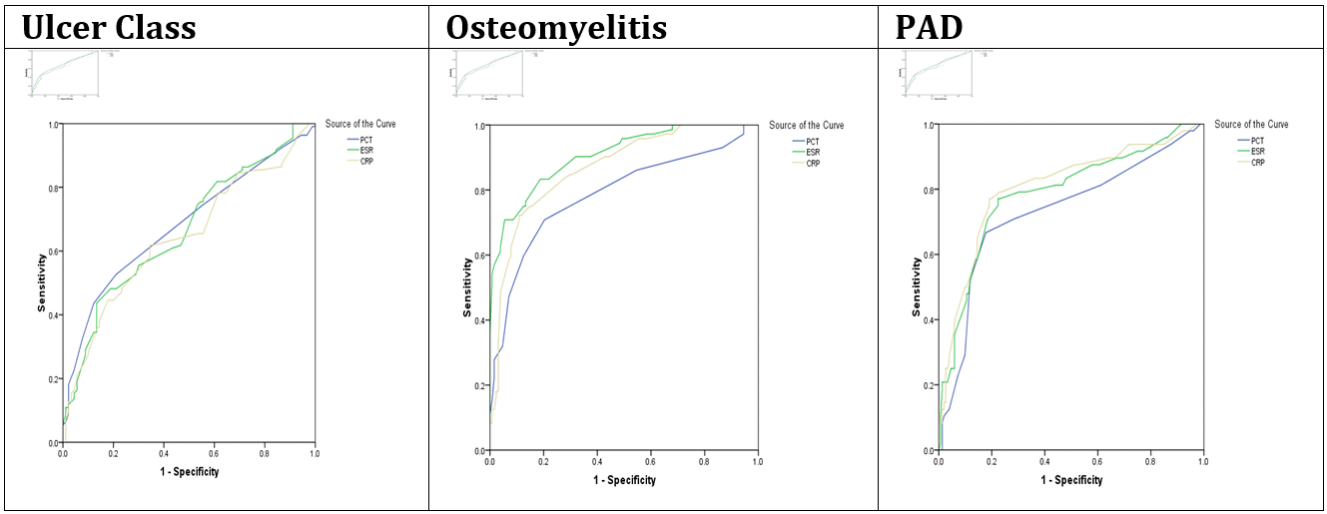
Area under the receiver operating characteristic (ROC) curve of pro-calcitonin (PCT), erythrocyte sedimentation rate (ESR), and C - reactive protein (CRP) in predicting the ulcer class, osteomyelitis, and peripheral arterial disease (PAD)

***Prediction of ulcer class***

The level of PCT, ESR and CRP were significantly higher in patients with class IV foot ulcer compared to those with class III (p<0.001, table 1). As summarized in table 2 and figure 1, ESR, CRP and PCT had moderate value for predicting class of diabetic foot ulcer according to ROC analysis.

***Prediction of osteomyelitis***

Patients with the evidence of osteomyelitis had significantly higher levels of PCT, ESR and CRP (table 1). The areas under the ROC curve of PCT, ESR and CRP in predicting osteomyelitis were 0.787, 0.869, and 0.907, respectively; showing high predictive value for each biomarker (table 2, figure 1). The best cutoff points and yielded sensitivity and sensitivity of PCT, ESR and CRP at that points in predicting osteomyelitis were 0.35 ng/ml (sensitivity of 86.1% and specificity of 45.3%), 56.5 mm/hours (sensitivity of 95.8% and specificity of 50.0%) and 44 mg/ml (sensitivity of 90.3% and specificity of 57.0%), respectively.

***Prediction of PAD***

The presence of PAD was significantly associated with increased levels of the three biomarkers. The areas under the ROC curve of PCT, ESR and CRP in predicting PAD were 0.733, 0.807 and 0.789, respectively (table 2, figure 1). The best cutoff values for PCT, ESR and CRP in predicting PAD were 0.45 (sensitivity of 70.8% and specificity of 71.7%), 61.5 (sensitivity of 83.3% and specificity of 52.0%) and 49 (sensitivity of 83.3% and specificity of 63.8%), respectively.

## Discussion

Based on the findings of the present study, although the accuracy of PCT, ESR and CRP in predicting the severity of diabetic foot ulcers was fair, the increase of the three parameters could predict the occurrence of osteomyelitis and PAD following diabetic food development with good accuracy and acceptable sensitivity. These three indicators seem to be beneficial in predicting wound infections rather than wound healing or classification. 

In the study by Korkmaz et al., the amount of CRP and ESR in patients with infectious ulcers was significantly higher than that of patients with non-infectious diabetic ulcers. Based on the analysis of the ROC curve, CRP had the highest capability for detecting infection, and the best cut-off point for CRP in predicting diabetic wound infection was 28 (15). This cut point, however, was 44 in our study for assessing the severity of the wound itself.

In the study by Park et al., PCT and CRP showed a high correlation with severity of diabetic ulcer infection, which was not consistent with our study. Also, PCT levels could be used to differentiate the existence of infection (16). This inconsistency can be due to the different methodology of our study as we were aiming to evaluate the general severity of wound, independent of infection, but in the Park et al. study, they have concentrated on the severity of wound infection rather than overall severity.

In the study by Massara et al., exploring the relationship between these markers and the onset of diabetic foot ulcer, showed that PCT and CRP markers had the highest diagnostic values for predicting the incidence of diabetic foot ulcers (13). 

In the study by Victoria et al., the sensitivity and specificity of PCT in prediction of diabetic foot ulcer infection were 81% and 90%, respectively. However, other markers were not associated with diabetic foot ulcer infection (17). In the study by Jonaidi et al. the best cutoff point, sensitivity and specificity for CRP and PCT to distinguish infection in 30 patients were reported as 7.1 mg/dL, 80%, 74%, and 0.21, 70% and 74%, respectively (18). A possible explanation for the visible differences between their study and ours could be the limited number of individuals in our study population, affecting the power of the results. 

Several studies have been conducted on the relationship of increase in ESR and CRP with predicting osteomyelitis in diabetic patients (19-21), but few studies have been done on the role of PCT in predicting osteomyelitis. Only in the study by Redman et al., the prominent role of PCT in periodontitis has been shown, which was shown to be even more potent than CRP (22). Also, there are few studies on the role of these markers in prediction of PAD occurrence. Increase in ESR and CRP was also associated with an increased risk of PAD occurrence (23).

In general, due to the confirmation of the role of PCT, ESR and CRP indicators in predicting the occurrence of osteomyelitis and PAD complications following diabetic foot infection, the three indicators can be used to design and introduce scoring systems that determine the severity or development of related complications of diabetic foot ulcer. 

***Limitations***

Small sample size and unknown duration of ulcers and infections were among the limitations of the present study. All patients were hospitalized, so the ulcers were in PEDIS 3 and 4 stages.

## Conclusion:

Although the role of PCT, ESR and CRP indices was not significant in predicting the severity of diabetic foot ulcer, the increase of the three parameters can predict the occurrence of two complications of osteomyelitis and PAD following the progression of diabetes with good accuracy and high sensitivity. 
